# Isolation of mucosa-associated microbiota dysbiosis in the ascending colon in hepatitis C virus post-sustained virologic response cirrhotic patients

**DOI:** 10.3389/fcimb.2024.1371429

**Published:** 2024-04-08

**Authors:** Yohei Midori, Takuto Nosaka, Katsushi Hiramatsu, Yu Akazawa, Tomoko Tanaka, Kazuto Takahashi, Tatsushi Naito, Hidetaka Matsuda, Masahiro Ohtani, Yasunari Nakamoto

**Affiliations:** ^1^ Second Department of Internal Medicine, Faculty of Medical Sciences, University of Fukui, Fukui, Japan; ^2^ Department of General Internal Medicine, Fukui-ken Saiseikai Hospital, Fukui, Japan

**Keywords:** mucosa-associated microbiota, hepatitis C, ascending colon, short-chain fatty acid, liver fibrosis, intestinal barrier, bile acid production

## Abstract

**Background:**

Achieving sustained virologic response (SVR) in patients infected with hepatitis C virus (HCV) reduces all-cause mortality. However, the mechanisms and risk factors for liver fibrosis and portal hypertension post-SVR remain incompletely understood. In the gut-liver axis, mucosa-associated microbiota (MAM) substantially influence immune and metabolic functions, displaying spatial heterogeneity at the anatomical intestinal site. We analyzed MAM composition and function to isolate the locoregional MAM involved in chronic liver disease progression in HCV post-SVR patients.

**Methods:**

We collected MAM samples from three intestinal sites (terminal ileum, ascending colon, and sigmoid colon) via brushing during colonoscopy in 23 HCV post-SVR patients and 25 individuals without liver disease (controls). The 16S rRNA of bacterial DNA in specimens collected with a brush and in feces was sequenced. The molecular expression of intestinal tissues and hepatic tissues were evaluated by quantitative real-time PCR.

**Results:**

In the post-SVR group, the microbial β-diversity of MAM, especially in the ascending colon, differed from the control group and was associated with liver fibrosis progression. In PICRUSt analysis, MAM in the ascending colon in the liver cirrhosis (LC) group showed compromised functions associated with the intestinal barrier and bile acid production, and FGF19 expression was markedly decreased in the terminal ileum biopsy tissue in the LC group. At the genus level, six short-chain fatty acid (SCFA)-producing bacterial genera, *Blautia*, *Alistipes*, *Roseburia*, *Agathobaculum*, *Dorea*, and *Pseudoflavonifractor* were reduced in the ascending colon of post-SVR LC patients.

**Conclusion:**

In patients of HCV post-SVR, we identified the association between the degree of liver fibrosis and dysbiosis of mucosa-associated SCFA-producing bacterial genera that may be related to intestinal barrier and bile acid production in the ascending colon.

## 1 Introduction

In, 2020, an estimated 57 million people worldwide were chronically infected with the hepatitis C virus (HCV) (Collaborators, 2022). The development of direct-acting antiviral (DAA) therapy has revolutionized clinical care (Martinello et al., 2023). Sustained virological response (SVR) has been linked to decreased mortality and morbidity in cirrhosis, decompensated liver disease, and hepatocellular carcinoma (HCC) (Martinello et al., 2023). Although several patients recover hepatic fibrosis when HCV is eliminated, in some cases, the improvement in liver fibrosis and portal hypertension is insufficient, and its mechanisms and risk factors are not completely understood (Lens et al., 2020; Rockey and Friedman, 2021).

Trillions of microorganisms in the human intestine are crucial for regulating health, and disruptions in gut microbial communities can cause disease (Hsu and Schnabl, 2023). In gut-liver axis, microbial dysbiosis contributes to the development of liver diseases such as metabolic dysfunction-associated fatty liver disease, alcoholic liver disease, and primary sclerosing cholangitis (Nakamoto et al., 2019; Albillos et al., 2020; Tilg et al., 2022; Hsu and Schnabl, 2023). Intestinal bacteria are categorized into two compartments: the luminal and the mucosa-adherent compartments, known as the mucosa-associated microbiota (MAM) (Sugiyama and Shiotani, 2021). Considering its proximity to the epithelium, MAM has a profound impact on the immune and metabolic health by directly affecting gut barrier function and the immune system (Juge, 2022). The taxonomic composition of MAM differs from that of feces, with the genera exhibiting spatial heterogeneity depending on the anatomical site of the intestinal tract influenced by local environmental–microbial interactions (Zhang et al., 2014; Matsumoto et al., 2019). Therefore, assessing and analyzing the locoregional MAM dysbiosis is crucial for understanding the exact involvement of host–microbiome interactions in liver disease.

Herein, we investigated the association between chronic liver disease (CLD)-related factors and MAM dysbiosis in HCV post-SVR patients. MAM from three intestinal sites (terminal ileum, ascending colon, and sigmoid colon) and feces were collected and analyzed for microbiota composition. The β-diversity of MAM in the ascending colon exhibited a significant correlation with hepatic fibrosis. In the MAM of the ascending colon in post-SVR liver cirrhosis (LC) patients, six bacterial species were reduced, and metagenomic functional analysis revealed impairment of the intestinal barrier and bile acid-producing function, which may have affected liver fibrosis progression.

## 2 Materials and methods

### 2.1 Patient enrollment

This study included 23 HCV post-SVR patients and 25 patients without liver disease who underwent total colonoscopy at our hospital between, 2019 and, 2021. **Tables 1** and **2** summarize the clinical characteristics of the patients. The LC group included patients with stage F4 fibrosis on liver biopsy at the time of treatment and those with shear wave velocity (SWV) > 1.86 m/s (LOGIQ S8, GE Healthcare, Milwaukee, WI, USA) in whom biopsy was not performed. The following potential enrollees were excluded: minors, patients with gastrointestinal infections, individuals with a predisposition to bleeding, patients with concurrent malignant tumors, including HCC, habitual alcohol consumption, and those with a history of antibiotic usage within the past two months (**Table 3**). All participants provided written informed consent. This study was conducted in accordance with the Declaration of Helsinki. The study design was approved by the Research Ethics Committee of the University of Fukui (registration number: 20190167).

**Table 1:** Characteristics of HCV post-SVR patients and controls.

Characteristics	HCV post-SVR (n=23)	Control (n=25)	p-value
Age (years) ^#^	71.9 ± 8.6	68.8 ± 13.2	ns *
Gender (male/female)	15/8	17/8	ns ^†^
BMI (kg/m^2^) ^#^	23.5 ± 3.9	24.2 ± 3.1	ns *
Platelet count (10^4^/μL) ^#^	16.3 ± 6.5	22.6 ± 4.7	<0.01 *
INR ^#^	1.05 ± 0.14	0.97 ± 0.07	ns *
AST (IU/L) ^#^	25.4 ± 11.6	24.0 ± 7.9	ns *
ALT (IU/L) ^#^	19.6 ± 13.2	23.5 ± 16.0	ns *
γGTP (IU/L) ^#^	56.2 ± 17.9	39.5 ± 31.2	ns *
ALP (IU/L) ^#^	94.7 ± 49.8	71.8 ± 21.5	ns *
Total Bilirubin (mg/dL) ^#^	0.9 ± 0.4	0.9 ± 0.4	ns *
Albumin (g/mL) ^#^	4.1 ± 0.5	4.2 ± 0.3	ns *
Child-Pugh grade (A/B/C)	21/2/0	–	–
ALBI grade (1/2a/2b/3)	15/6/1/1	–	–
FIB-4 index ^#^	3.1 ± 1.7	1.8 ± 1.0	<0.01 *
Type IV collagen-7S (ng/mL) ^#^	6.3 ± 4.0	–	–
M2BPGi (C.O.I) ^#^	3.1 ± 3.6	–	–
Chronic hepatitis (CH)/Liver cirrhosis (LC)	12/11	–	–
Biopsy, F; staging (F1-3/F4), n=19	9/10	–	–
SWV of patients without biopsy (<1.86/>1.86), n=4	3/1	–	–
PPI users, number (%)	8 (34.8)	6 (24.0)	ns ^†^
Antibiotics users, number (%)	0 (0.0)	0 (0.0)	ns ^†^
Probiotics users, number (%)	0 (0.0)	1 (4.0)	ns ^†^
Antiviral therapy			
Direct acting antivirals, number (%)	16 (69.6)	–	–
Interferon plus ribavirin, number (%)	6 (26.1)	–	–
Other, number (%)	1 (4.3)	–	–
Past history of HCC, number (%)	10 (43.5)	0 (0.0)	<0.01 ^†^
Current status of HCC, number (%)	0 (0.0)	0 (0.0)	ns ^†^
Time of follow-up (years) ^#^	5.8 ± 4.8	–	–

ALBI, albumin–bilirubin; ALP, alkaline phosphatase; ALT, alanine aminotransferase; AST, aspartate aminotransferase; BMI, body mass index; FIB-4 index, fibrosis- 4 index, gGTP, gamma-glutamyl transpeptidase; HCC, hepatocellular carcinoma; HCV, hepatitis C virus; INR, international normalized ratio; ns, not significant; PPI, proton pump inhibitor; SVR, sustained virologic response; SWV, shear wave velocity. ^#^Continuous variables presented as mean ± standard deviation. *Mann–Whitney U test. ^†^Fisher’s exact test.

**Table 2:** Characteristics of hepatitis C virus post-sustained virologic response patients with chronic hepatitis and cirrhosis.

Characteristics	HCV post-SVR CH (n=12)	HCV post-SVR LC (n=11)	p-value
Age (years) ^#^	74.5 ± 6.7	69.0 ± 9.5	ns*
Gender (male/female)	7/5	8/3	ns ^†^
BMI (kg/m^2^) ^#^	22.5 ± 2.2	24.7 ± 4.8	ns *
Platelet count (10^4^/μL) ^#^	20.0 ± 6.3	12.3 ± 3.9	<0.01 *
INR ^#^	1.00 ± 0.09	1.11 ± 0.16	ns *
AST (IU/L) ^#^	21.6 ± 4.3	29.5 ± 15.2	ns *
ALT (IU/L) ^#^	16.2 ± 6.2	23.4 ± 17.2	ns *
γGTP (IU/L) ^#^	22.8 ± 13.6	39.8 ± 17.8	<0.01 *
ALP (IU/L) ^#^	67.9 ± 10.9	124.0 ± 58.5	<0.01 *
Total Bilirubin (mg/dL) ^#^	0.9 ± 0.4	1.0 ± 0.4	ns *
Albumin (g/mL) ^#^	4.3 ± 0.4	3.8 ± 0.5	ns *
Child-Pugh grade (A/B/C)	12/0/0	9/2/0	–
ALBI grade (1/2a/2b/3)	10/2/0/0	5/4/1/1	–
FIB-4 index ^#^	2.2 ± 0.9	4.1 ± 2.0	<0.01 *
Type IV collagen-7S (ng/mL) ^#^	3.7 ± 0.9	8.9 ± 4.3	<0.01 *
M2BPGi (C.O.I) ^#^	1.1 ± 0.5	5.0 ± 4.2	<0.01 *
PPI users, number (%)	2 (16.7)	6 (54.5)	ns ^†^
Antibiotics users, number (%)	0 (0.0)	0 (0.0)	ns ^†^
Probiotics users, number (%)	0 (0.0)	0 (0.0)	ns ^†^
Antiviral therapy			
Direct acting antivirals, number (%)	7 (58.3)	9 (81.8)	–
Interferon plus ribavirin, number (%)	5 (41.7)	1 (9.1)	–
Other, number (%)	0 (0.0)	1 (9.1)	–
Past history of HCC, number (%)	3 (25.0)	8 (72.7)	<0.01 ^†^
Current status of HCC, number (%)	0 (0.0)	0 (0.0)	ns ^†^
Time of follow-up (years) ^#^	7.1 ± 5.2	4.3 ± 3.9	ns *

ALBI, albumin–bilirubin; ALP, alkaline phosphatase; ALT, alanine aminotransferase; AST, aspartate aminotransferase; BMI, body mass index; CH, chronic hepatitis; FIB-4 index, fibrosis- 4 index, gGTP, gamma-glutamyl transpeptidase; HCC, hepatocellular carcinoma; HCV, hepatitis C virus; INR, international normalized ratio; LC, liver cirrhosis; PPI, proton pump inhibitor; SVR, sustained virologic response; SWV, shear wave velocity. ^#^Continuous variables presented as mean ± standard deviation. *Mann–Whitney U test. ^†^Fisher’s exact test.

**Table 3:** Inclusion and exclusion criteria.

Inclusion criteria	Exclusion criteria
1) HCV post-SVR patients or patients without liver disease who underwent total colonoscopy between, 2019 and, 2021	1) Minors
	2) Gastrointestinal infections
	3) Predisposition to bleeding
2) Patients with consent for sample collection	4) Concurrent malignant tumors
3) Observation to the terminal ileum is possible	5) Habitual alcohol consumption
	6) History of antibiotic usage within the past two months

### 2.2 Sample collection

Total colonoscopy was performed following the usual colonoscopy preparation using polyethylene glycol. The mucosal surface was gently scraped using a sterile cytology brush (TeleMed Systems Inc., MA, USA) and for each respective case, samples were collected from terminal ileum, ascending colon, and sigmoid colon. Additionally, stool samples were collected on the day of examination. In cases with obtained consent, mucosal and liver biopsy tissues were collected during HCV treatment and preserved in RNA later (Thermo Fisher Scientific, MA, USA) at −20°C.

### 2.3 DNA extraction and 16S rRNA sequencing

Double-stranded DNA (dsDNA) was extracted from each sample using the NucleoSpin DNA Stool Kit (MACHEREY-NAGEL, Düren, Germany). The measurements of DNA concentration were performed using Qubit dsDNA HS assay (Thermo Fisher Scientific), and 5 ng of DNA was used for the first PCR targeting the V3-V4 region of the 16S rRNA gene with Universal primers 341F (5′-TCGTCGGCAGCGTCAGATGTGTATAAGAGACAGCCTACGGGNGGCWGCAG-3′) and 805R (5′-GTCTCGTGGGCTCGGAGATGTGTATAAGAGACAGGACTACHVGGGTATCTAATCC-3′) (Thermo Fisher Scientific). The PCR products were confirmed using electrophoresis, purified using AMPure XP beads (Beckman Coulter, CA, USA), and subjected to index PCR using the Nextera XT index kit (Illumina, San Diego, CA, USA). Subsequent bead purification was conducted and the final library concentration was diluted to 4 nM for normalization. The DNA was denatured by adjusting the final denatured DNA concentration to 6 pM using 25% PhiX spike-in. Sequencing was performed using a MiSeq Reagent v2 kit with a 250-bp paired-end strategy according to the manufacturer’s instructions (Illumina) (Muyzer et al., 1993; Inoue et al., 2016).

### 2.4 Bioinformatics analysis

Paired-end FASTQ files obtained from sequencing were uploaded from the Basespace hub, an Illumina cloud database, to the Ezbiocloud (ChunLab, Inc., Seoul, Republic of Korea). Using the EzBioCloud pipeline, all raw sequencing data were re-analyzed for each 16S-based microbial taxonomic profile (MTP). Microbiome profiling was conducted using the MTP platform of the application, with the 16S database version PKSSU.4.0 (Yoon et al., 2017). The MTP sets were constructed by grouping the individual MTPs. The MTP sets were comparatively analyzed after normalizing the gene copy numbers. Beta (β) diversity was evaluated using Jensen–Shannon divergence and generalized UniFrac distances, and the results were visualized using principal coordinate analysis (PCoA). We subsequently performed taxonomic biomarker analysis based on linear discriminant analysis (LDA) effect size (LEfSe) to identify differentially distributed taxa between MTP sets that could potentially be used as microbial biomarkers. Taxa that demonstrated significant results in the relative abundance (p < 0.05) and LEfSe analysis (LDA score > 3.00) were considered significant biomarkers (Kim et al., 2023).

### 2.5 Functional metagenome prediction

The EzBioCloud 16S-based MTP pipeline employed the phylogenetic investigation of communities by reconstruction of unobserved states (PICRUSt) algorithm to estimate the functional profiles of the microbiome identified using 16S rRNA sequencing. Raw sequencing reads were processed using the EzBioCloud 16S microbiome pipeline with default parameters and discriminating reads encountered in the reference database. Functional abundance profiles of MAM were annotated using bioinformatics analyses. This specifically involved multiplying the vector of gene counts for each operational taxonomic unit (OTU) by the abundance of that OTU in each sample, using the Kyoto Encyclopedia of Genes and Genomes (KEGG) orthology and pathway database. The predicted metagenomic profiles were categorized into clusters of KEGG pathways and compared between the post-SVR CH and LC groups (Ferreira et al., 2018; Tango et al., 2020).

### 2.6 RNA extraction from biopsy specimens and subsequent real-time PCR

Intestinal mucosa or liver biopsy tissues were homogenized using Micro Smash (TOMY, Tokyo, Japan), and total RNA was extracted using an RNeasy Mini Kit (Qiagen, Hilden, Germany). The cDNA was synthesized from RNA using a High Capacity RNA-to-cDNA Kit (Applied Biosystems, Foster City, CA, USA) after quantification and purity assessment using a Nanodrop (Thermo Fisher). Quantitative real-time PCR (qRT-PCR) was conducted using a StepOnePlus real-time PCR system (Applied Biosystems) with a TaqMan probe (**Table 4**) (Thermo Fisher). The ΔΔCt comparative threshold method was used for analyzing the expression levels of the target genes. GAPDH was considered an internal control.

**Table 4:** Primers used in this study.

Gene	Species	Dye	Company	Catalogue No.
FGF19	Human	FAM	Applied Biosystems	Hs00192780_m1
IL-8	Human	FAM	Applied Biosystems	Hs00174103_m1
TGF-β1	Human	FAM	Applied Biosystems	Hs00998133_m1
GAPDH	Human	FAM	Applied Biosystems	Hs02786624_g1

### 2.7 Statistical analysis

Permutational multivariate analysis of variance (PERMANOVA) and the Kruskal–Wallis test were used to analyze the statistical differences in beta diversity and for functional analysis, respectively. Both analyses were performed using Ezbiocloud. The Mann–Whitney and Spearman’s correlation tests were conducted using Prism (version 9; GraphPad, GraphPad Software Inc.). Statistical significance was set at p-values < 0.05.

## 3 Results

### 3.1 MAM in post-SVR LC patients exhibit dysbiosis at the genus level

**Figure 1:**
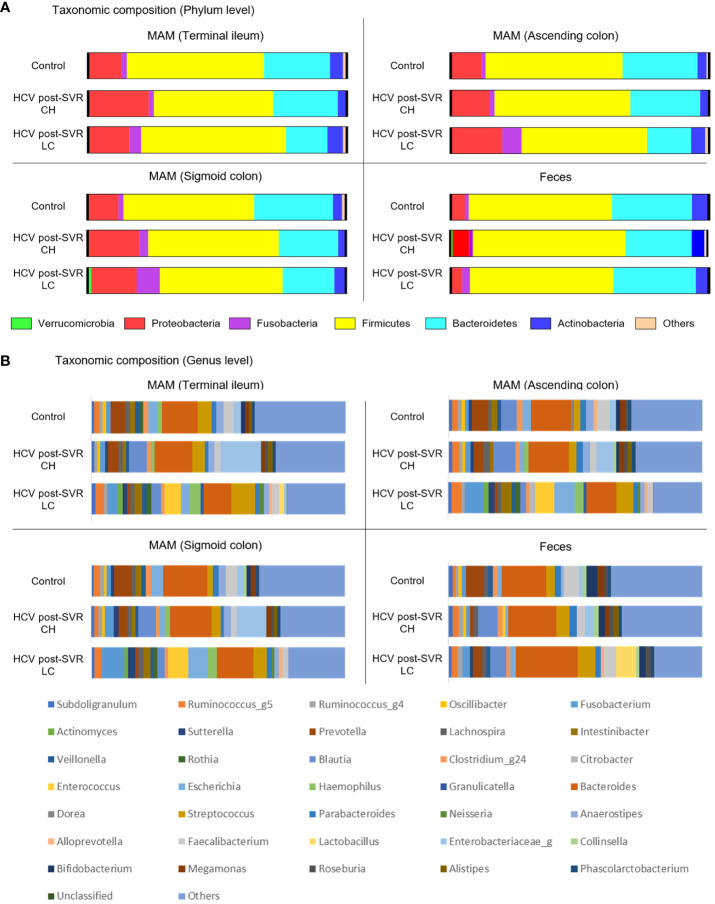
The relative abundance of bacteria at the phylum and genus levels of the mucosa-associated microbiota (MAM) in the terminal ileum, ascending colon, and sigmoid colon as well as feces of the control and patients infected with HCV after sustained virologic response (SVR) chronic hepatitis (CH) and liver cirrhosis (LC). The bar graphs represent the **(A, B)** relative amounts of bacterial composition at the phylum **(A)** and genus levels **(B)** of the MAM in the terminal ileum, ascending colon, and sigmoid colon as well as feces from the control (n=25), HCV SVR-CH (n=12), and HCV SVR-LC (n=11) groups. Labels except for “others” indicate the phylum or genera at the average relative abundance (≥1%) in at least one sampling site. CH, chronic hepatitis; HCV, hepatitis C virus; LC, liver cirrhosis; MAM, mucosa-associated microbiota; SVR, sustained virologic response.

Microbiota composition was analyzed in the MAM of the terminal ileum, ascending and sigmoid colon, and feces of patients infected with HCV who achieved an SVR. We compared the data between patients with non-cirrhotic (chronic hepatitis; CH) and cirrhotic (liver cirrhosis; LC) liver diseases and controls. Comparing patients with CH and LC revealed no significant differences between the groups at either sampling site at the phylum level by the Mann–Whitney U test (**Figure 1A**). At the genus level, patients with LC exhibited a distinctly different composition from patients with CH and the control group in MAM in the terminal ileum, ascending colon, and sigmoid colon (**Figure 1B**; **Table 5**). These results indicate that microbiota dysbiosis was observed at the genus level in the MAM of post-SVR LC patients.

**Table 5:** Genera with significant differences in comparison between HCV post-SVR CH and HCV post-SVR LC on each site.

Site	Genera	Relative abundance in Control	Relative abundance in HCV post-SVR CH	Relative abundance in HCV post-SVR LC	p-value*	p-value*
		(mean ± SD)	(mean ± SD)	(mean ± SD)	Ctrl vs LC	CH vs LC
Terminal ileum	*Bilophila*	0.22 ± 0.25	0.24 ± 0.24	0.10 ± 0.16	0.198	0.042
	*Atopobium*	0.16 ± 0.27	0.08 ± 0.11	0.52 ± 0.72	0.074	0.022
	*Anaerotignum*	0.16 ± 0.16	0.30 ± 0.29	0.07 ± 0.10	0.048	0.016
	*Coprobacter*	0.06 ± 0.12	0.13 ± 0.24	0.01 ± 0.01	0.619	0.031
	*Holdemania*	0.03 ± 0.04	0.05 ± 0.06	0.02 ± 0.05	0.144	0.029
Ascending colon	*Blautia*	6.07 ± 3.80	8.72 ± 5.76	2.27 ± 2.31	0.002	0.002
	*Alistipes*	1.12 ± 1.13	1.58 ± 1.69	0.34 ± 0.47	0.009	0.022
	*Dorea*	1.07 ± 0.99	0.77 ± 0.60	0.20 ± 0.42	0.003	0.031
	*Roseburia*	0.82 ± 0.91	1.38 ± 1.03	0.42 ± 0.58	0.154	0.013
	*Sporobacter*	0.63 ± 0.97	0.13 ± 0.22	0.06 ± 0.11	0.001	0.016
	*Pseudoflavonifractor*	0.50 ± 0.31	0.73 ± 0.63	0.24 ± 0.38	0.003	0.022
	*Agathobaculum*	0.44 ± 0.30	0.70 ± 0.54	0.17 ± 0.29	0.004	0.003
	*Anaerotignum*	0.27 ± 0.38	0.28 ± 0.27	0.09 ± 0.11	0.035	0.019
	*Coprobacter*	0.07 ± 0.16	0.14 ± 0.25	0.01 ± 0.02	0.159	0.001
	*Atopobium*	0.06 ± 0.07	0.02 ± 0.03	0.39 ± 0.77	0.272	0.038
Sigmoid colon	*Agathobaculum*	0.48 ± 0.31	0.70 ± 0.68	0.23 ± 0.33	0.071	0.045
	*Sporobacter*	0.37 ± 0.47	0.17 ± 0.26	0.06 ± 0.11	0.001	0.017
	*Anaerotignum*	0.24 ± 0.34	0.27 ± 0.26	0.12 ± 0.16	0.236	0.029
	*Anaerococcus*	0.03 ± 0.05	0.01 ± 0.04	0.08 ± 0.13	0.583	0.014
Feces	*Alistipes*	0.92 ± 1.20	1.69 ± 1.49	0.34 ± 0.41	0.118	0.018
	*Parasutterella*	0.72 ± 1.07	0.26 ± 0.37	0.06 ± 0.12	0.026	0.024
	*Klebsiella*	0.42 ± 1.10	0.49 ± 1.54	0.02 ± 0.03	0.461	0.022
	*Odoribacter*	0.16 ± 0.19	0.20 ± 0.18	0.08 ± 0.13	0.135	0.011
	*Anaerotignum*	0.15 ± 0.19	0.28 ± 0.37	0.08 ± 0.11	0.311	0.031
	*Coprobacter*	0.05 ± 0.13	0.14 ± 0.16	0.01 ± 0.02	0.086	0.009

CH, chronic hepatitis; HCV, hepatitis C virus; LC, liver cirrhosis; Ctrl, control; SD, standard deviation; SVR, sustained virologic response. * Mann–Whitney U test.

### 3.2 The β-diversity of MAM in the ascending and sigmoid colon differs in HCV post-SVR patients

**Figure 2:**
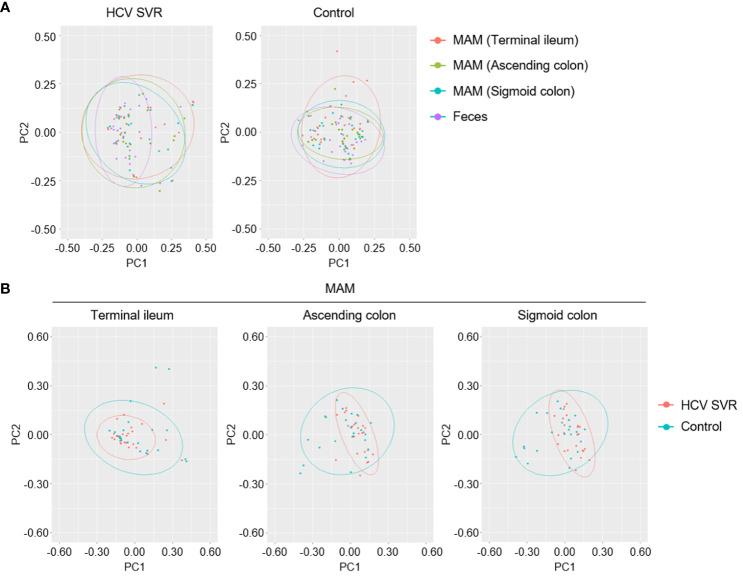
Weighted principal coordinate analysis of β-diversity at the genus level of MAM in HCV post-SVR and control groups. **(A, B)** Weighted principal coordinate analysis (PCoA) plots of β-diversity at the genus level of the microbial community. **(A)** Comparison of the β-diversity of MAM of each sampling site: terminal ileum, ascending colon, and sigmoid colon as well as feces in the HCV post-SVR (n=23) and control groups (n=25). **(B)** Comparison of β-diversity of the HCV post-SVR and control groups of MAM at each sampling site: terminal ileum, ascending colon, and sigmoid colon. The statistical significance among the two groups was determined using permutational multivariate analysis of variance (PERMANOVA).

In the HCV post-SVR and the control group, microbial β-diversity at the genus level for MAM at each sampling site and feces was conducted using Principal Coordinates Analysis (PCoA) based on Jensen-Shannon divergence. PERMANOVA test revealed a substantial difference in the β-diversity of MAM of the feces and the terminal ileum, ascending colon, and sigmoid colon in the HCV post-SVR group (**Figure 2A**; **Table 6**). In the control group, the β-diversity of MAM in the terminal ileum was different from that in feces. The β-diversity of MAM in the ascending and sigmoid colon exhibited significant differences in the HCV post-SVR and control groups for each sampling site (**Figure 2B**; **Table 7**). These results indicate that the microbial β-diversity of MAM in the post-HCV SVR group, especially in the ascending colon and sigmoid colon, differed from that in the control group.

**Table 6:** PERMANOVA test for the β-diversity of MAM collected at each site and feces in HCV post-SVR patients and controls.

PERMANOVA test for the β-diversity of each site in HCV post-SVR patients			
Group 1	Group 2	pseudo-F	p-value
Feces	Terminal ileum	3.103	0.008
	Ascending colon	2.335	0.030
	Sigmoid colon	3.137	0.004
Terminal ileum	Ascending colon	0.423	0.882
	Sigmoid colon	0.442	0.862
Ascending colon	Sigmoid colon	0.061	0.997
PERMANOVA test for the β-diversity of each site in controls			
Group 1	Group 2	pseudo-F	p-value
Feces	Terminal ileum	2.817	0.013
	Ascending colon	1.662	0.121
	Sigmoid colon	1.837	0.084
Terminal ileum	Ascending colon	0.735	0.619
	Sigmoid colon	1.161	0.304
Ascending colon	Sigmoid colon	0.116	0.991

**Table 7:** PERMANOVA test of the β-diversity of MAM per intestinal site in HCV post-SVR patients and the controls.

Site	pseudo-F	p-value
Terminal ileum	1.098	0.358
Ascending colon	2.061	0.040
Sigmoid colon	2.886	0.001

### 3.3 The β-diversity of MAM in the ascending colon is associated with liver fibrosis progression

**Figure 3:**
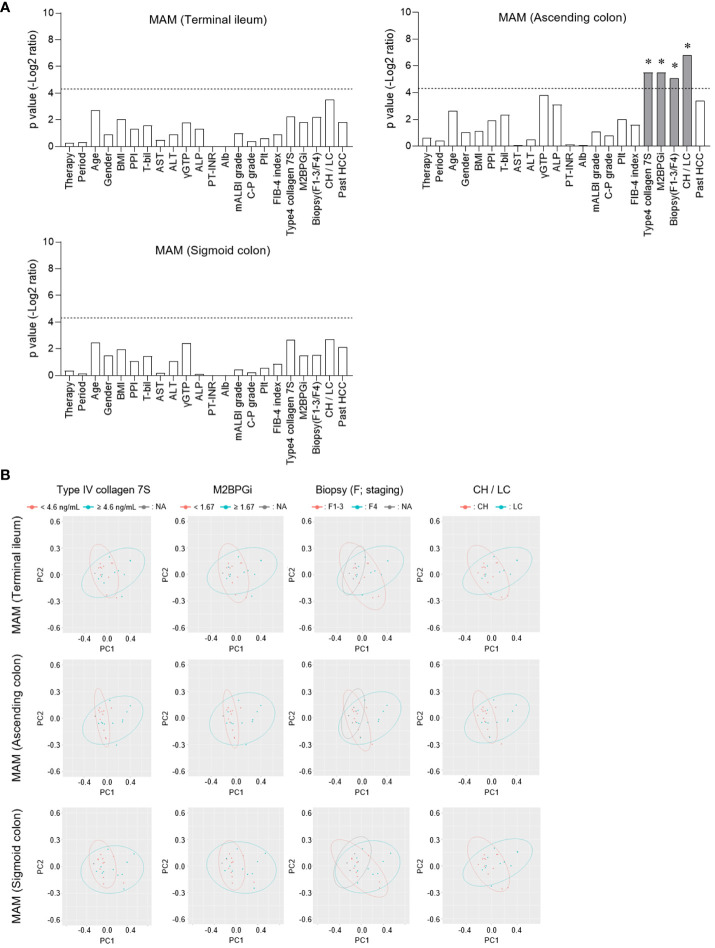
Comparison of the correlation between β-diversity of mucosa-associated microbiota (MAM) and chronic liver disease-related factors at each sampling site. **(A, B)** In patients of HCV post-SVR (n=23), each clinical parameter was divided into two groups at the stated cutoff values, and the β-diversity of MAM of each sampling site (terminal ileum, ascending colon, and sigmoid colon) was compared. **(A)** Among the two groups, p values (−log2 ratio) were determined using permutational multivariate analysis of variance (PERMANOVA). The dotted line indicates p = 0.05. *p < 0.05. **(B)** Weighted principal coordinate analysis (PCoA) plots of β-diversity at the genus level of MAM in the terminal ileum, ascending colon, and sigmoid colon were drawn in two divided groups according to the type IV collagen 7S, M2BPGi, Biopsy, and CH/LC parameters.

We performed a PERMANOVA test for each MAM collection site (terminal ileum, ascending, and sigmoid colon) to evaluate the CLD-related factors associated with the microbial β-diversity of MAM (**Figure 3A**). Factors related to liver fibrosis, such as type IV collagen 7S, M2BPGi, and biopsy (F1-3/F4, CH/LC), were significantly correlated in the MAM of the ascending colon (**Figures 3A, B**). The association between CLD-related factors and MAM in the terminal ileum or the sigmoid colon was statistically insignificant. Thus, the β-diversity of MAM in the ascending colon is associated with liver fibrosis progression in HCV post-SVR patients.

### 3.4 MAM in the HCV SVR-LC group may have impaired intestinal barrier and bile acid production

**Figure 4:**
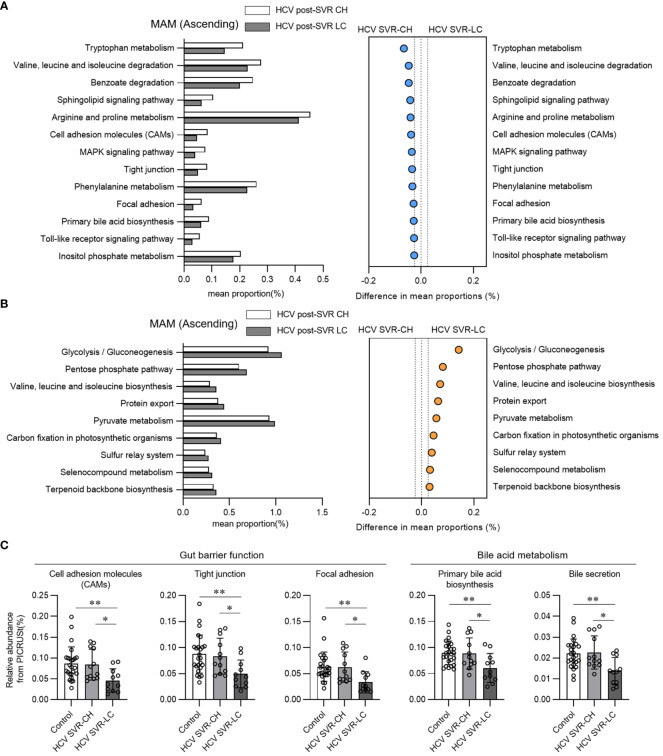
Functional analysis in predictive metagenomics using phylogenetic investigation of communities by reconstruction of unobserved states in the mucosa-associated microbiota (MAM) of the ascending colon of patients with HCV post-sustained virologic response (SVR) chronic hepatitis (CH) and liver cirrhosis (LC). **(A, B)** Relative abundance of the predicted functions according to the Kyoto Encyclopedia of Genes and Genomes (KEGG) pathways of level 3 in the MAM of the ascending colon of the HCV post-SVR CH and LC groups. Pathways with statistically significant differences (p<0.05) between the two groups and relative differences > 0.025 were extracted. **(A)** Pathways that are proportionally decreased in the HCV SVR-LC group compared to the CH group. **(B)** Pathways that are proportionally increased in the HCV SVR-LC group compared to the CH group. **(C)** The relative abundance of MAM in the ascending colon in the control, post-HCV CH, and LC groups in the extracted five KEGG pathways. Data represent the means ± standard deviation. Kruskal–Wallis test. *p < 0.05, **p < 0.01.

We performed a functional analysis of predictive metagenomics using PICRUSt in HCV post-SVR patients to evaluate the function of MAM in the ascending colon in liver fibrosis. We compared the relative proportion levels associated with the KEGG pathway in the MAM of the ascending colon in the HCV SVR-CH and LC groups (**Figures 4A, B**). Cell adhesion molecules, tight junctions, focal adhesions, and primary bile acid biosynthesis were the pathways that were proportionally downregulated in the LC group. Pathways related to the intestinal barrier function (cell adhesion molecules, tight junctions, and focal adhesion) and bile acid production (primary bile acid biosynthesis and bile secretion) were significantly decreased in the MAM of the HCV SVR-LC group compared to those of the control and HCV SVR-CH groups (**Figure 4C**). Thus, MAM in the ascending colon in the HCV post-SVR LC group may have impaired intestinal barrier and bile acid production.

### 3.5 Tissue expression of the bile acid-related and fibrosis-related molecules associated with liver fibrosis

**Figure 5:**
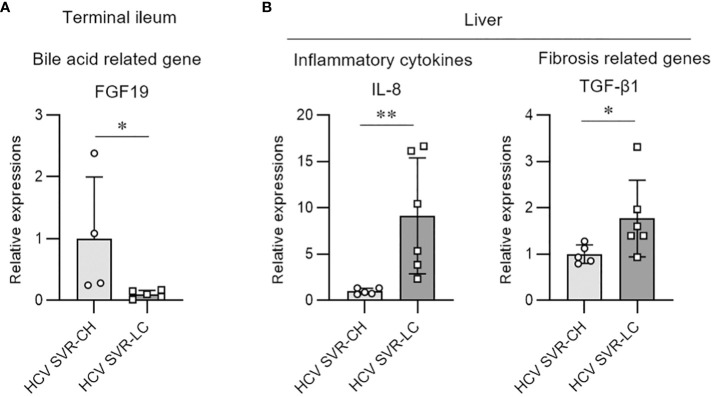
Molecular expression of bile acid and fibrosis-related genes in the intestine and hepatic tissues of the patients with HCV post-sustained virologic response liver cirrhosis and chronic hepatitis groups. Quantitative real-time PCR analysis of the mRNA expression in the biopsy samples from the terminal ileum **(A)** and liver tissue **(B)**. Data represent means ± standard deviation. Mann–Whitney U test. *p < 0.05, **p < 0.01.

We evaluated the molecular expression of bile acid signaling in intestinal tissues and fibrosis-related genes in the hepatic tissues of the HCV post-SVR LC and CH groups. FGF19 expression, a downstream signaling molecule of bile acids and farnesoid X receptor (FXR), was markedly decreased in the terminal ileum biopsy tissue of the SVR LC group compared to that in the CH group (**Figure 5A**). In the liver tissue, IL-8 and TGF-β1 expression, cytokines associated with the progression of liver fibrosis, were markedly increased in the SVR LC group compared to that in the CH group (**Figure 5B**). These findings suggest that the SVR LC group exhibits decreased expression of the intestinal bile acid-related signaling molecules and increased expression of the liver fibrosis-related cytokines.

### 3.6 The bacterial genus of MAM in the ascending colon is associated with liver fibrosis progression in HCV post-SVR patients

**Figure 6:**
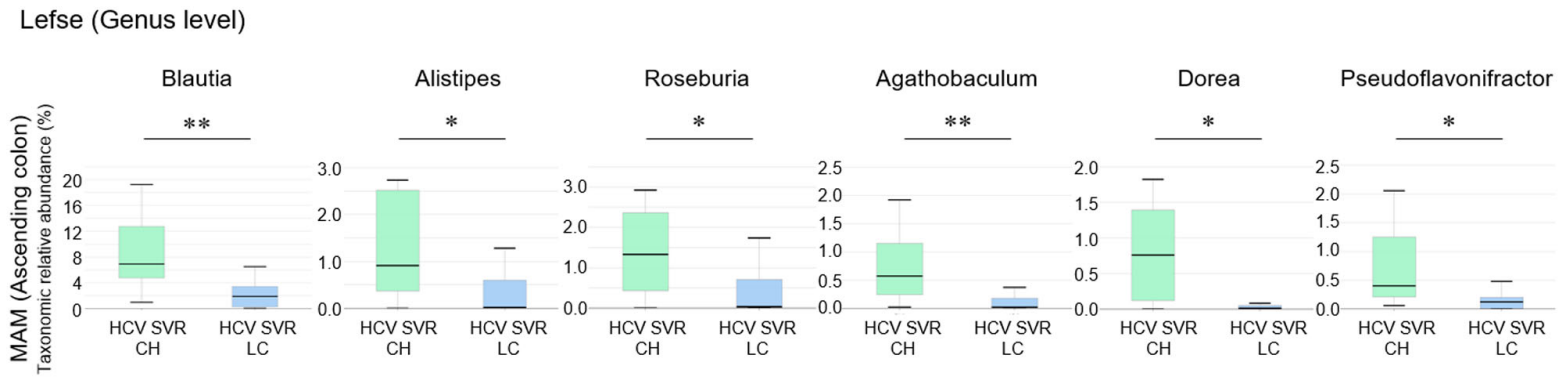
The relative composition of the taxonomic level in mucosa-associated microbiota (MAM) of the ascending colon between the HCV post-sustained virologic response (SVR) liver cirrhosis (LC) and chronic hepatitis (CH) groups. Genera with significant differences in relative abundance between HCV post-SVR CH and LC patients (p<0.05, LDA score>3). Data represent means ± standard deviation. Mann–Whitney U test. *p < 0.05, **p < 0.01. LDA, linear discriminant analysis.

We compared the differences in the relative composition at the taxonomic level in the MAM of the ascending colon between the HCV post-SVR LC and CH groups using LEfSe. The genera *Blautia* (p=0.002, LDA score 4.52), *Alistipes* (p=0.022, LDA score 3.75), *Roseburia* (p=0.013, LDA score 3.67), *Agathobaculum* (p=0.003, LDA score 3.44), *Dorea* (p=0.031, LDA score 3.42), and *Pseudoflavonifractor* (p=0.022, LDA score 3.36) were significantly decreased in the MAM of ascending colon in HCV post-SVR LC group (**Figure 6**). All six genera were short-chain fatty acid (SCFA)-producing bacteria.

### 3.7 The correlation between the genus composition of MAM in the ascending colon and liver fibrosis-related factors in HCV post-SVR patients

**Figure 7:**
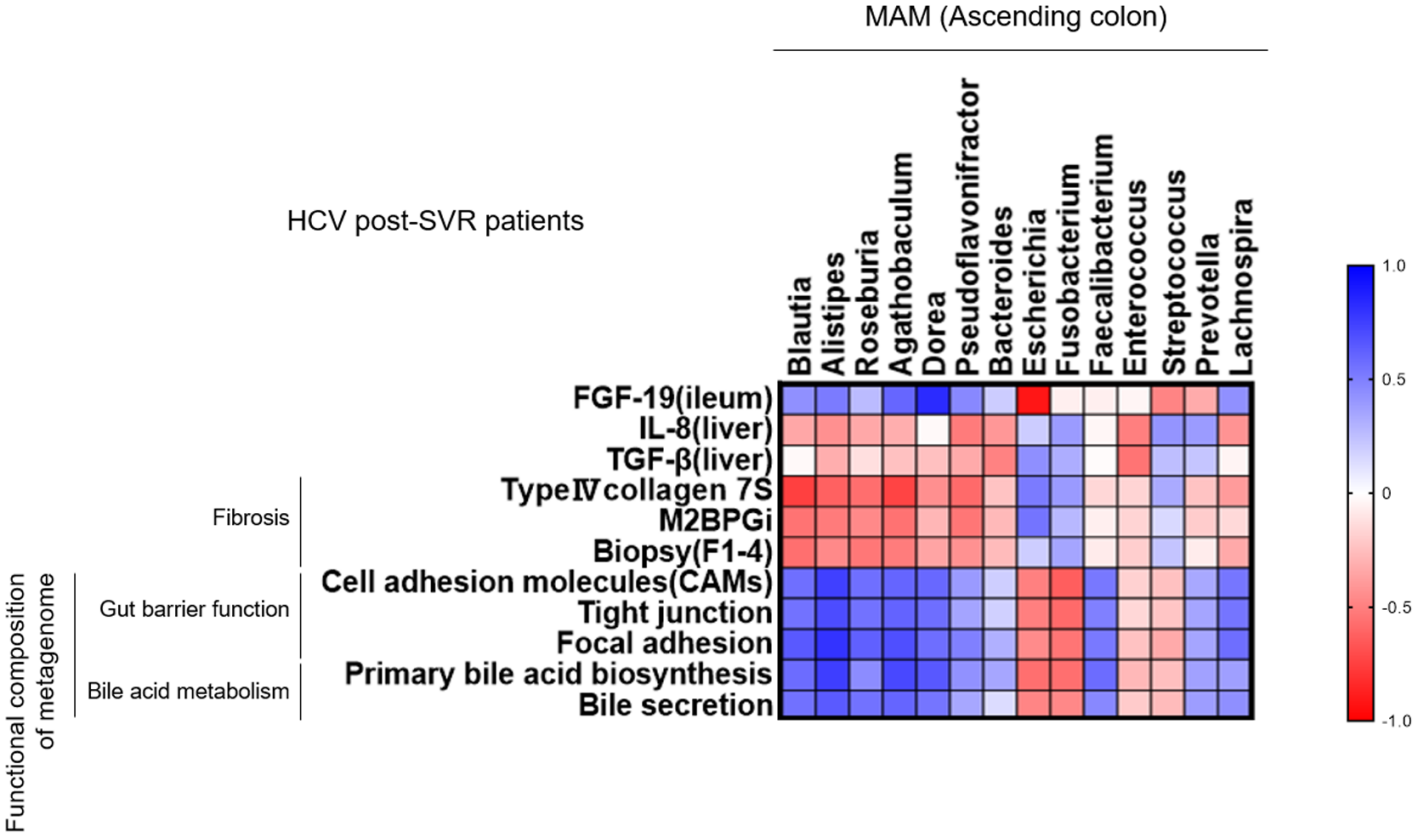
Correlation between the genus composition of mucosa-associated microbiota (MAM) in the ascending colon and liver fibrosis-related factors in HCV post-sustained virologic response (SVR) patients. Heatmap diagram representing the correlation between the genera extracted from the MAM of the ascending colon and liver fibrosis markers, molecular expression in the intestinal and liver tissues, and the function of metagenomics in HCV post-SVR LC patients.

We analyzed the correlation between the genera composition of MAM in the ascending colon and liver fibrosis markers, molecular expression in the intestinal and liver tissues, and the function of metagenomics in HCV post-SVR patients (**Figure 7**). Six genera (*Blautia*, *Alistipes*, *Roseburia*, *Agathobaculum*, *Dorea*, and *Pseudoflavonifractor*) of MAM in the ascending colon were negatively correlated with the degree of fibrosis of liver tissue and serum liver fibrosis markers, such as type IV collagen-7S and M2BPGi, compared with the other dominant genera. Additionally, they positively correlated with the functional gene sets related to the intestinal barrier and bile production as well as FGF19 expression in the terminal ileum tissue. Conversely, they exhibited a negative correlation with IL-8 and TGF-β1 expression in the liver tissue. Thus, in post-HCV SVR cases, the composition of the six genera in the MAM of the ascending colon is associated with the hepatic fibrosis degree and bacterial flora function related to liver fibrosis.

## 4 Discussion

In HCV post-SVR patients, MAM were collected from three intestinal sites (terminal ileum, ascending colon, and sigmoid colon) and feces. The microbial β-diversity of MAM in the post-SVR group, especially in the ascending and sigmoid colon, differed from that in the control group. Additionally, the β-diversity of MAM in the ascending colon is associated with liver fibrosis progression. PICRUSt analysis revealed that MAM in the ascending colon in the post-SVR LC group had impaired functions related to the intestinal barrier and bile acid production. Six SCFA-producing bacterial genera were reduced in the ascending colon of post-SVR LC patients, and their composition was correlated with the progression of liver fibrosis.

MAM on the colonic epithelial surface plays an important role in maintaining health by training the immune system, promoting epithelial growth, and regulating mucus production (Wang et al., 2010; Hsu and Schnabl, 2023). MAM is the focus of attention in liver disease. Bajaj et al. reported a greater association between MAM and hepatic encephalopathy than microbiota of feces in patients with cirrhosis (Bajaj et al., 2012). Haraguchi et al. reported the decreased diversity of in the MAM of cirrhotic patients is risk factor for minimal hepatic encephalopathy (Haraguchi et al., 2019). Moreover, studies on intestinal site-specific MAM showed a correlation between rectal MAM composition and alcoholic cirrhosis (Shen et al., 2021) and an association between ileal MAM and primary biliary cholangitis (Kitahata et al., 2021). These findings suggest the significance of analyzing MAM at multiple specific sites in elucidating the pathogenesis of patients with liver disease.

Spatial heterogeneity of the mucosal microbiota along the human intestinal tract has been observed (Hong et al., 2011; Zhang et al., 2014). MAM structure and function are influenced by the changes in mucus, pH, oxygen levels, and bile acid-mediated signals (Albillos et al., 2020; Juge, 2022). The pH and mucin composition increase and luminal contents are dehydrated toward the rectum (Bown et al., 1974). SCFA, lactic acid, and ethanol concentrations were higher in the proximal colon and decreased toward the distal colon (Zhang et al., 2014). These local environment-microbes or host-microbe interactions are major driving forces in spatial heterogeneity formation of the mucosal microbiota along the intestinal tract (Zhang et al., 2014). These reports support the finding that the bacterial composition of MAM differs by site in HCV post-SVR patients. This emphasizes the importance of analyzing MAM at specific colonic sites rather than in fecal samples.

Herein, six SCFA-producing bacteria*—Blautia*, *Alistipes*, *Roseburia*, *Agathobaculum*, *Dorea*, and *Pseudoflavonifractor—*were reduced in the MAM of the ascending colon in post-SVR LC patients compared to post-SVR CH patients. Intestinal bacteria produce SCFAs, which perform various biological functions such as homeostasis of the gut–liver axis by maintaining intestinal permeability and regulating immunity, lipogenesis, and gluconeogenesis (Jin et al., 2019; Cao et al., 2023). HCV infection has been reported to decreases the fecal bacterial composition of *Blautia* (Chuaypen et al., 2023), *Alistipes* (Sultan et al., 2021), *Dorea* (Chuaypen et al., 2023; Yang et al., 2023), and *Pseudoflavonifractor* (Heidrich et al., 2018). Regarding the microbial composition of feces, *Roseburia* decreases in patients with cirrhosis (Chen et al., 2011) and *Agathobaculum* decreases in patients with non-alcoholic fatty liver disease (Ahn et al., 2019). Wellhöner et al. reported that among the HCV patients treated with DAA, fecal microbial diversity increased in patients without cirrhosis at SVR 24/48, but not in those with cirrhosis (Wellhoner et al., 2021). Interestingly, in HCV post-SVR patients with advanced liver fibrosis, dysbiosis and six SCFA-producing bacteria—*Blautia*, *Alistipes*, *Roseburia*, *Agathobaculum*, *Dorea*, and *Pseudoflavonifractor*— were more significant in the MAM of the ascending colon than in the fecal luminal microbiota.

The gut–liver axis refers to the bidirectional relationship between the gut and its microbiota, and the liver. This reciprocal interaction is established by the portal vein and the liver feedback route of bile acid (BA) and antibody secretion to the intestine (Albillos et al., 2020). FXR are nuclear receptors of BAs (Zhu et al., 2023). FGF 19, the main FXR target gene in the gut, is secreted into the portal blood upon BA stimulation (Avila and Moschetta, 2015). Bile flow in the intestine is markedly reduced in end-stage liver disease (Ikegami and Honda, 2018). In cirrhosis, dysbiosis of the gut microbiota and decreased FXR signaling disrupt tight junctions in the gut epithelium. This allows the influx of lipopolysaccharides into the liver, triggering cytokine production (e.g., IL-8 and TGF-β1) in the liver and initiating a cascade that exacerbates hepatitis and promotes liver fibrosis (Albillos et al., 2020; Liu et al., 2022). These findings support our results, indicating compromised MAM function and altered gene expression of liver fibrosis-related cytokines in the hepatic and intestinal tissues in post-SVR LC patients.

This study has several limitations. First, this was a single-center study with a small sample size. This is because the evaluation of brush-collected MAM and feces was limited to those who underwent colonoscopy. Second, microbial changes in patients with HCV infection and SVR were not analyzed because bacterial specimens were not collected before DAA or IFN therapy. Third, various foods may be associated with improved diet and functional optimization of the microbiota; however, we did not consider individual dietary information in this study (Tilg et al., 2022). Future analysis of larger numbers of patients and microbial change in HCV post-SVR patients will provide more robust evidence regarding the impact of MAM. This is expected to further elucidate the mechanism of liver fibrosis and its potential therapeutic applications.

In conclusion, among the MAM in the terminal ileum, ascending colon, and sigmoid colon, as well as feces, only β-diversity of MAM in the ascending colon substantially correlated with liver fibrosis in patients with HCV after SVR. In HCV post-SVR patients, we identified an association between the degree of liver fibrosis and dysbiosis of mucosa-associated SCFA-producing bacterial genera, which may be related to the intestinal barrier and bile acid production in the ascending colon.

## Data availability statement

The datasets presented in this study can be found in online repositories. The names of the repository/repositories and accession number(s) can be found below: https://www.ncbi.nlm.nih.gov/, PRJNA1070593.

## Ethics statement

The studies involving humans were approved by the Research Ethics Committee of the University of Fukui (registration number: 20190167). The studies were conducted in accordance with the local legislation and institutional requirements. The participants provided their written informed consent to participate in this study.

## Author contributions

YM: Conceptualization, Data curation, Formal analysis, Investigation, Methodology, Software, Visualization, Writing – original draft. TNo: Conceptualization, Data curation, Formal analysis, Investigation, Visualization, Writing – original draft. KH: Conceptualization, Data curation, Formal analysis, Investigation, Writing – original draft. YA: Conceptualization, Data curation, Formal analysis, Investigation, Writing – original draft. TT: Conceptualization, Data curation, Formal analysis, Investigation, Writing – original draft. KT: Conceptualization, Data curation, Formal analysis, Investigation, Writing – original draft. TNa: Conceptualization, Data curation, Formal analysis, Investigation, Writing – original draft. HM: Conceptualization, Data curation, Formal analysis, Investigation, Writing – original draft. MO: Conceptualization, Data curation, Formal analysis, Investigation, Writing – original draft. YN: Conceptualization, Data curation, Formal analysis, Funding acquisition, Investigation, Methodology, Project administration, Supervision, Visualization, Writing – original draft, Writing – review & editing.
